# Post-Polymerization Modification of Fluoropolymers via UV Irradiation in the Presence of a Photoacid Generator

**DOI:** 10.3390/polym15030493

**Published:** 2023-01-17

**Authors:** Anastasia Nika, Christina Gkioka, Fotini Machairioti, Panayiotis Bilalis, Jiaxi Xu, Katarzyna Gajos, Kamil Awsiuk, Panagiota Petrou, Margarita Chatzichristidi

**Affiliations:** 1 Industrial Chemistry Laboratory, Department of Chemistry, National and Kapodistrian University of Athens, Zografou, 15771 Athens, Greece; 2 Immunoassay/Immunosensors Lab, INRaSTES, NCSR “Demokritos”, Aghia Paraskevi, 15310 Athens, Greece; 3 Polymer Synthesis Laboratory, KAUST Catalysis Center, Physical Sciences and Engineering Division, King Abdullah University of Science and Technology (KAUST), Thuwal 23955, Saudi Arabia; 4 M. Smoluchowski Institute of Physics, Jagiellonian University, Łojasiewicza 11, 30-348 Kraków, Poland

**Keywords:** fluoropolymers, bio-functional surfaces, optical lithography, hydrophilicity, biosensor

## Abstract

Fluorinated polymers have unique wettability and protein adsorption properties. The site-specific alteration of these properties could expand their application to different research areas. In this work, a fluorinated homopolymer and two of its copolymers with 4-vinylbenzyl glycidyl ether (VBGE) are synthesized by free radical polymerization. The produced polymers are then used to develop resist formulations by the addition of a photoacid generator. Films of these formulations are exposed to ultraviolet radiation through a binary mask and heated to create the pattern. It is found that the water contact angle values of the exposed films areas are reduced compared to those of the unexposed ones, with the exception of pentafluorophenyl methacrylate (PFMA) homopolymer film. This is attributed to the reaction of the epoxy groups creating x-links and producing hydroxyl groups and the cleavage of the pentafluorophenyl group from the ester group leading to carboxylic acid groups. Both modifications on the exposed areas are verified by FTIR spectroscopy and ToF-SIMS analysis. In addition, the biomolecules adsorption ability of the exposed area is increasing 10–15 times compared to the unexposed one for the PFMA homopolymer and the PFMA/VBGE 1:1 copolymer. Thus, the proposed polymers and patterning procedure could find application to spatially directed immobilization of biomolecules and/or cells onto a surface for both biosensing and tissue engineering purposes.

## 1. Introduction

Polymeric materials, due to their attractive physicochemical and mechanical properties, play an essential role in many applications in the modern world, among them in biological applications such as tissue engineering and regenerative medicine [1,2], bio-arrays [3], lab-on-a-chip devices [4], and much more. Most of these applications use polymers to benefit not only from the material’s bulk properties but also from the ease of their surface modification, which enables tuning the surface properties according to the requirements of each particular application [5,6,7,8,9,10,11]. There are three general methods for surface modification of polymers: physicochemical, mechanical, and biological [10,12].

Physicochemical methods are related to alterations in the chemical composition of the surfaces employing techniques such as active-gas or vapor treatment for deposition of a thin film onto the surface [10,13] or alteration of surface texture [14,15,16], exposure to radiation (ultraviolet [13,17,18,19,20] or extreme ultraviolet [7]), treatment of the surface with solvents [21,22,23], bulk phase desorption or combination of those treatments [10,13,18,24]. Mechanical methods are related to the alternation of the surface texture through mechanical roughening/polishing (e.g., sandblasting) [25] and micromanipulation (e.g., using AFM, STM probes). Biological methods employ biomolecules to modify the substrate; this can be achieved either through physical adsorption of biomolecules (e.g., proteins, peptides) or chemical conjugation of biomolecules to surface groups [18]. Among those surface modification methods, UV treatment is one of the simplest and easiest to implement since a UV lamp can be found in most labs. There are additional advantages when this technique is combined with that of lithography, via exposure to UV radiation through a binary mask, to modify some surface areas selectively while leaving the rest unaltered. The Argitis group has already demonstrated this technique by exposing an epoxy resist surfaced at 254 nm through a mask. The exposed areas of the film had a much higher binding ability of reactive biotin than the unexposed ones, thereby creating a high-resolution patterning of biomolecules [17]. Amongst the different categories of polymers, fluoropolymers exhibit many unique properties like excellent chemical resistance and resistance to environmental corrosion [14], high-temperature stability, low dielectric constant, and low water sorption [26]. Due to these properties, many researchers are investigating the use of these materials in bio-applications after appropriate modification of the polymeric surface by introducing functional groups or changing their physicochemical properties at specific areas [27,28,29,30]. To this end, Heitz et al. modified polytetrafluoroethylene (PTFE) surfaces with exposure at wavelengths below 200 nm in an ammonia atmosphere. They observed that the F atoms of the polymer were substituted from H atoms, produced from the dissociation of the exposed ammonia molecules, rendering the surface more hydrophilic compared to the untreated one. This modification enhanced cell adhesion and proliferation onto the treated versus the untreated surfaces [13,18].

With those examples in mind, this work aims to modify fluoropolymers in specific areas via UV irradiation and study how this affects their protein adsorption properties. The fluoropolymer selected was poly(pentafluorophenyl methacrylate) (PPFMA), which according to the literature could be processed after polymerization to cleave the pentafluorophenyl group and enable the reaction of the active ester group with different amine-containing compounds [21,22,23,31,32,33]. In the current work, the postpolymerization modification was attempted by adding a photoacid generator in the polymer solution prior to its spin coating onto silicon wafers. The sample was then exposed to UV through a mask and subsequent heating to achieve cleavage of the pentafluorophenyl group from the polymer chain only in the exposed areas. Apart from the PPFMA homopolymer, copolymers with 4-vinylbenzyl glycidyl ether (VBGE) were synthesized using different molar ratios of the two homopolymers and the changes in their chemical composition, water contact properties, and biomolecule binding ability upon UV exposure and heating were determined.

## 2. Materials and Methods

### 2.1. Materials and Instruments

Sodium hydride (60% dispersion in mineral oil), glycidol, 1-(chloromethyl)-4-vinylbenzene, and 2,2′-azobis(2-methylpropionitrile) solution (AIBN, 0.2 M in toluene), and propylene glycol methyl ether acetate (PGMEA) were purchased from Sigma-Aldrich (Darmstadt, Germany). Ethyl L-lactate, 99%, was from Alfa Aesar (Waltham, MA, USA). All solvents were used as purchased without further purification, unless otherwise specified. The photoacid generator (PAG) used in the resist formulations was triphenyl sulphonium hexafluoro-antimonate salt (TPS-103; Midori Kagaku Co., Ltd., Tokyo, Japan). Sulfo-succinimidyl-6-(biotinamido)hexanoate (EZ-Link™ Sulfo-NHS-LC-Biotin) and streptavidin labeled with AlexaFluor^®^ 456 (AF456) were purchased from Thermo Fisher Scientific (Waltham, MA, USA). Bovine serum albumin (BSA) was purchased from Acros Organics (Geel, Belgium) and was biotinylated following a previously published protocol [28].

Nuclear Magnetic Resonance (^1^H NMR and ^13^C{^1^H} NMR) measurements were recorded on a Bruker AVANCE III-400 MHz instrument (Bruker, Billerica, MA, USA). Size Exclusion Chromatography (SEC) measurements were performed at 50 °C with DMF (containing 1 M LiBr, Sigma-Aldrich) as an eluent at a flow rate of 1.0 mL min^−1^ in an Agilent 1260 infinity system (Agilent Technologies, Santa Clara, CA, USA) equipped with a 1200 HPLC pump, an Optilab T-rEX RI detector, and three 7.8 × 300 mm columns (Styragel^®^ HT 2, 3, and 4) for poly(VGBE) homopolymer and the copolymers. Size exclusion chromatography (SEC) measurements were carried out at 35 °C with THF (Sigma-Aldrich) as an eluent at a flow rate of 1.0 mL min^−1^ in a Viscotek GPCmax VE2001 system (Malvern Panalytical, Malvern, UK) equipped with PSS columns (Styragel^®^ HR 3, 4 and 5). The dispersity (M_w_/M_n_, Ð) was determined by conventional SEC analysis using a calibration curve obtained with polystyrene standards for poly(PFMA) homopolymer. SEC samples in THF were prepared by dissolving 2 mg/mL solutions in THF and filtered through 0.22 µm Teflon filters before injection. The polymeric films were exposed at 254 nm using an 8 Watt UV lamp (UVLMS-38 EL Series 3UV, UVP, Analytik Jena US, Upland, CA, USA). Fourier Transform Infrared (FTIR) spectra of the films were received using a Spectrum One FT-IR Spectrometer from PerkinElmer (Waltham, MA, USA). Due to Si absorption peaks at 1115-1065 cm^−1^ (Si–OSi), the spectra of a clean Si wafer were taken as the background.

Water contact angles were measured by applying 5 μL droplets of deionized water using the Digidrop Contact Angle Measurement System from GBX (Dublin, Ireland). Time of Flight-Secondary Ion Mass Spectroscopy (ToF-SIMS, ION-TOF GmbH, Münster, Germany) analysis was conducted using an ion dose density of about 1012 ions/cm^2^, corresponding to static mode, and applying a current of about 0.5 pA. A low energy electron flood gun was used for charge compensation. Positive ion high mass resolution TOF-SIMS spectra were acquired from 10 non-overlapping 100 μm × 100 μm (applied resolution was 128 × 128 points) areas of each sample. Mass calibration was performed with H^+^, H_2_^+^, CH^+^, C_2_H_2_^+^, and C_4_H_5_^+^ peaks. A minimal mass resolution (m/∆m) > 8000 at C_4_H_5_^+^ was obtained.

### 2.2. Synthesis of 4-Vinylbenzyl Glycidyl Ether (VBGE)

**Figure 1:**
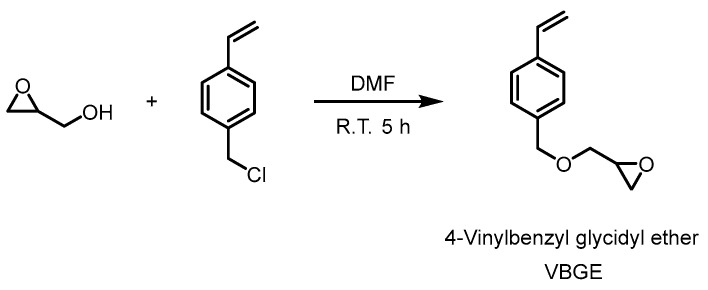
The synthesis of 4-vinylbenzyl glycidyl ether (VGBE).

4-Vinybenzyl glycidyl ether was synthesized according to the reaction of Figure 1. Sodium hydride (60% dispersion in mineral oil, 4.0 g, 0.1 mol) was suspended in DMF (200 mL) at 0 °C followed by addition of glycidol (6.6 mL, 0.1 mol). After stirring at 0 °C for 1 h, 1-(chloromethyl)-4-vinylbenzene (7 mL, 0.05 mol) was added, and the mixture was further stirred at room temperature for 5 h. The mixture was cooled down to 0 °C and excess diethyl ether was added to dilute the reaction mixture. Saturated aqueous ammonium chloride was added to quench the reaction. The resulting white solid was filtered off and the aqueous layer was extracted three times with diethyl ether. The combined organic layers were dried over sodium sulfate and the solvent was removed in vacuo. The residue was purified by column chromatography (PE/EA, 10:1) to afford a faint yellow liquid 4-vinylbenzyl glycidyl ether (7.3 g, 77% yield). The ^1^H NMR spectra and ^13^C{^1^H} NMR spectra are given in Figure S1.

### 2.3. Synthesis of 4-Vinylbenzyl Glycidyl Ether (VBGE) and Pentafluorophenyl Methacrylate (PFMA) Homopolymers

Solutions of VBGE (0.024 mol, 4.56 g) or PFMA (0.024 mol, 6.0 g) and AIBN (4 × 10^−5^ mol, 0.2 mL) in 6 mL DMF were prepared in Schlenk tubes and degassed using five freeze-pump-thaw cycles to remove oxygen. The Schlenk tubes were then placed in oil baths at 70 °C to initiate polymerization. After completion, the reaction mixture was diluted by CH_2_Cl_2_ and the product polymers were precipitated into methanol to remove the residual unreacted monomers. The white precipitates were collected via centrifugation, and dried at 40 °C in a vacuum oven. ^1^H NMR spectra and SEC profile for poly(VBGE) and poly(PFMA) are given in Figures S2 and S3, respectively.

### 2.4. Synthesis of 4-Vinylbenzyl Glycidyl ether (VBGE) and Pentafluorophenyl Methacrylate (PFMA) Copolymers

**Figure 2:**
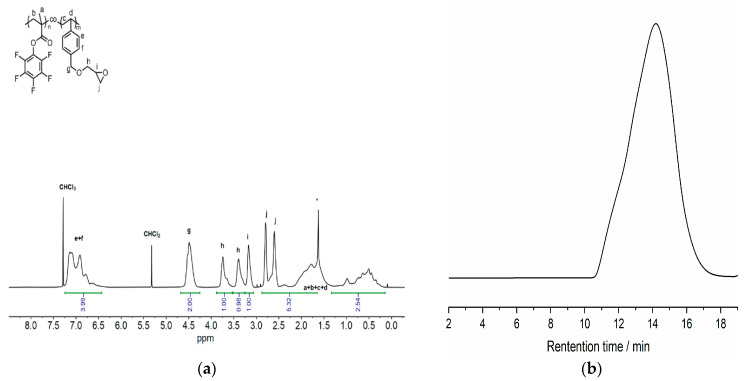
(**a**) ^1^H NMR (400 MHz, CDCl_3_) spectrum of poly(PFMA-co-VBGE) 1:1 copolymer ([PFMA]/[VBGE] = 1/0.97). Asterisk (*) represents H_2_O. (**b**) SEC trace of poly(PFMA)-co-poly(VBGE) 1:1 copolymer (M_n,SEC_ = 93.5 kg mol^−1^, M_w_/M_n_ = 3.26) received using as eluent DMF at a flow rate of 1.0 mL min^−1^ with an Agilent 1260 infinity system equipped with a 1200 HPLC pump, an Optilab T-rEX RI detector, and three 7.8 × 300 mm columns (Styragel^®^ HT 2, 3, and 4).

Solutions of: (a) VBGE (0.006 mol, 1.14 g), PFMA (0.018 mol, 4.5 g), and AIBN (4 × 10^−5^ mol, 0.2 mL) in 6 mL DMF and (b) VBGE (0.012 mol, 2.28 g), PFMA (0.012 mol, 3.0 g) and AIBN (4 × 10^−5^ mol, 0.2 mL) in 6 mL DMF, were prepared in Schlenk tubes to obtain the [PFMA]/[ VBGE] ratios of 3:1 and 1:1, respectively. The solutions were degassed using five freeze-pump-thaw cycles to remove oxygen. The reaction Schlenk tubes were then placed in oil baths at 70 °C to initiate polymerization. After completion, the reaction mixtures were diluted with CH_2_Cl_2_ and the product polymers were precipitated into methanol to remove the residual unreacted monomers. The white precipitates were collected via centrifugation, and dried at 40 °C in a vacuum oven. In Table 1 the copolymers feed ratio and characteristics are given. In Figure 2, the NMR spectra as well as the SEC profile of the PFMA-VBGE 1:1 copolymer are provided, whereas for PFMA-VBGE 3:are given in Figure S4 of the Supplementary material.

### 2.5. Preparation of Resist Modified Substrates and Photolithography Procedure

Solutions with a concentration of 5% *w*/*w* were prepared for the two copolymers and the VBGE homopolymer in PGMEA, and in THF for the PPFMA homopolymer. After overnight stirring, the photoacid generator (PAG) triphenyl sulfonium hexafluoro-antimonate was added in each polymer solution at a concentration of 5% *w*/*w*, with respect to the polymer, and the resulting solution was stirred overnight at room temperature. The resist formulations were filtered using a CA 45-μm pore filter (Macherey-Nagel GmbH & Co., Düren, Germany) prior to application onto Si wafers using a spin-coater at 3000 rpm for 60 s with 500 rpm/s ramping. The resist-coated substrates were then baked at 110 °C for 3 min using a hot plate, and exposed using a UV lamp at 254 nm (8 Watt, 1 mW/cm^2^) for 40 or 60 min, followed by a final baking step at 110 °C for 3 min on a hotplate. FTIR spectra and contact angle measurements were carried out in all samples.

### 2.6. Biomolecule Adsorption Protocol

The procedure for evaluating the biomolecule adsorption properties of the films both prior and after exposure to UV was as follows: a 50 μg mL^−1^ biotinylated-BSA solution in 0.05 M phosphate buffer, pH 7.4, was applied to the surface and incubated for 1 h at room temperature. Then, the surfaces were washed with distilled water, dried on a N_2_ stream, and immersed in a 1% *w*/*v* BSA solution in the same buffer (blocking solution) for 1 h. After washing and drying as previously, the samples were incubated with a 2 μg mL^−1^ AlexaFluor 546 labeled streptavidin solution in blocking solution for 30 min. Then, they were washed with 0.05 M phosphate buffer, pH 7.4, containing 0.05% *v*/*v* Tween 20, and with distilled water, and dried on a N_2_ stream. Fluorescence images were acquired using an Axioskop 2 plus epifluorescence microscope (Carl Zeiss AG, Jena, Germany) equipped with a MicroPublisher 3.3 RTV CCD camera (Teledyne QImaging, Surrey, BC, Canada). The fluorescence intensity on the areas where the biomolecules were immobilized was determined quantitatively using the Image Pro-Plus software (Media Cybernetics Co., Rockville, MD, USA). The net fluorescence values were determined by subtracting the values obtained by areas of the same surface coated with blocking protein only.

## 3. Results and Discussion

**Figure 3:**
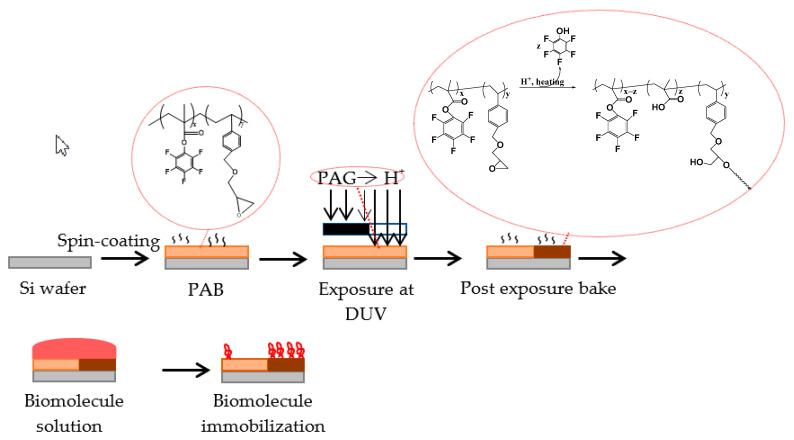
Schematic representation of the procedure followed for surface coating with the resist solution, exposure to UV, development, and biomolecule immobilization. The unexposed area of the film is colored with light brown whereas the exposed area is colored with dark brown.

A schematic presentation of the procedure followed to prepare the substrates is provided in Figure 3. Initially, the polymers resist formulations were spin-coated on Si wafers. Then, the films were post applied baked (PAB) followed by exposure at 254 nm through a binary mask. During exposure, acid is produced from the photoacid generator, and in the thermal treatment that follows (post exposure bake, PEB), hydrolysis of the ester group and cleavage of pentafluorophenyl group from the polymer chain takes place. Simultaneously, the epoxy rings open and x-link reactions take place [12,34,35]. In this way, the modified substrates have two areas, the unexposed ones that are rich in fluorine-atoms (light brown area in Figure 3) and the exposed x-linked areas possessing hydroxyl and carboxylic acid groups (dark brown area in Figure 3).

### 3.1. Surface Characterization

#### 3.1.1. FTIR Spectra

**Figure 4:**
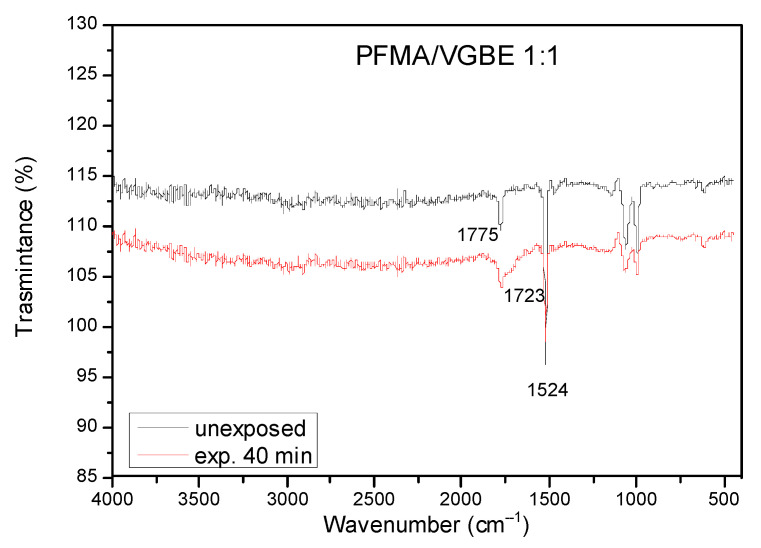
FTIR spectra of the unexposed (black line) and the 40 min exposed to UV area (blue line) of a PFMA/VBGE 1:1 copolymer resist film spin-coated on a Si wafer.

First, the polymer resist modified substrates were characterized by FTIR spectroscopy. In Figure 4, the FTIR spectra of the PFMA/VBGE 1:1 copolymer film before and after exposure and PEB are shown. The spectrum of the unexposed film shows a peak at 1775 cm^−1^ corresponding to the C=O group of the ester. This peak is decreased at the spectrum of the exposed film and another peak at 1723 cm^−1^ appears that is attributed to the C=O group of the carboxylic acid. In addition, the peak at 1524 cm^−1^, due to the phenyl group, is decreased at the exposed film spectrum compared to that of the unexposed one. Similar changes are observed at the FTIR spectra of the other polymers (see Supplementary Material Figure S5). The reduction in the peaks corresponding to the C=O group of the ester and the phenyl of the pentafluorophenyl group at the spectra of the exposed films coupled with the appearance of the peak of the carboxyl group indicate the partial cleavage of the pentafluorophenyl group from the polymers film upon UV exposure. The removal of the side pentafluorophenyl group is expected to result in changes in the water contact angle of the exposed versus the unexposed areas, rendering the film surface more hydrophilic. This effect combined with the presence of –COOH groups is expected to promote the physisorption of proteins in the exposed areas. In this way, the film will have two areas—the unexposed with low physisorption of the proteins and the exposed area with much higher protein adsorption.

#### 3.1.2. Contact Angle Measurements

The water contact angle of the different polymer resist films prior to and after exposure to UV light was measured for all samples in three different areas and the mean value of the measurements is given in Table 2.

From the results presented in Table 2, it can be concluded that the water contact angle of the exposed areas of all copolymers tested changes upon exposure to UV light as expected. In particular, the exposed areas of the film become more hydrophilic as the water contact angle drops about 10–15 degrees compared to the unexposed ones. The hydrophilicity change of the exposed polymeric films is mostly driven by the formation of hydroxyl groups during the x-linking reaction of the epoxy groups. In addition, the moderate change in the water contact angle value of the copolymers after UV exposure conforms to the fact that a big percentage of the pentafluorophenyl group remains in the copolymers film after exposure. On the other hand, there is no change in the water contact angle value of the PFMA homopolymer after exposure, probably due to the high content of fluoro group on its surface. In order to further investigate the water contact angle change of the two copolymers, in the SI section (Table S1) a table is given with the contact angles of the two copolymers after immersion of their unexposed and exposed films in different solution for 1h. To gain further insight into the chemical changes occurring upon exposure of the polymers to UV light, analysis by ToF-SIMS was also contacted.

#### 3.1.3. ToF SIMS Spectra

ToF-SIMS analysis was employed to examine the cleavage of the pentafluorophenyl group and the opening of the epoxy group upon exposure of the polymeric films to UV light. For that purpose, the relative intensities of the signals corresponding to two ions were selected: C_6_F_5_^−^ that is characteristic of the pentafluorophenyl group of PFMA, and C_3_H_5_O^+^ as the characteristic group of the epoxy ring of VGBE.

**Figure 5:**
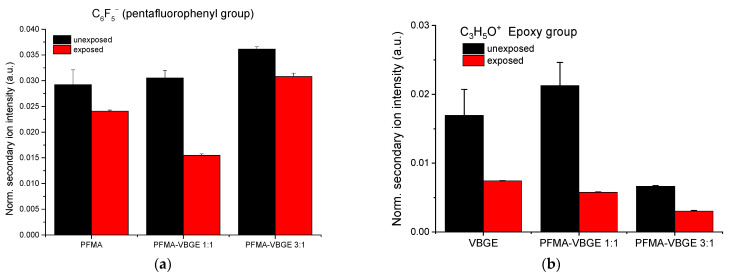
(**a**) Normalized ToF-SIMS signal intensities corresponding to the C_6_F_5_^−^ ion of unexposed (black columns) or exposed areas (red columns) of PFMA homopolymer in THF, PFMA/VBGE 1:1 copolymer and PFMA/VBGE 3:1 copolymer in PGMEA. (**b**) Normalized ToF-SIMS signal intensities corresponding to the C_3_H_5_O^+^ ion of unexposed (black columns) or exposed areas (red columns) of VBGE homopolymer in THF, PFMA/VBGE 1:1 copolymer and PFMA/VBGE 3:1 copolymer in PGMEA.

Figure 5a clearly shows that the pentafluorophenyl group is partially cleaved from the surface of the polymers as the intensity of the characteristic ion is reduced in the exposed vs. the unexposed areas. The highest difference in the intensity of the characteristic ion is observed for the PFMA/VBGE 1:1 copolymer, probably due to the lower content of pentafluorophenyl group on the film, thus leaving more mobility for the acid to find the ester group. Regarding the intensity of the ion characteristic for the epoxy group, as shown in Figure 5b, it is significantly reduced in the exposed areas of both the homopolymer and the copolymers, indicating the opening of the epoxy group and thereby confirming the anticipated changes in the polymers side chains due to UV exposure (see Figure 3). In the SI section (Figure S6), the normalized ToF-SIMS signal intensities corresponding to the H_3_O^+^ ion—corresponding to the hydroxyl groups of the unexposed or exposed areas for all the polymeric films—are given, showing that a higher epoxy ring percentage in the unexposed film leads to higher hydroxyl groups in the exposed film.

### 3.2. Biomolecule Adsorption

**Figure 6:**
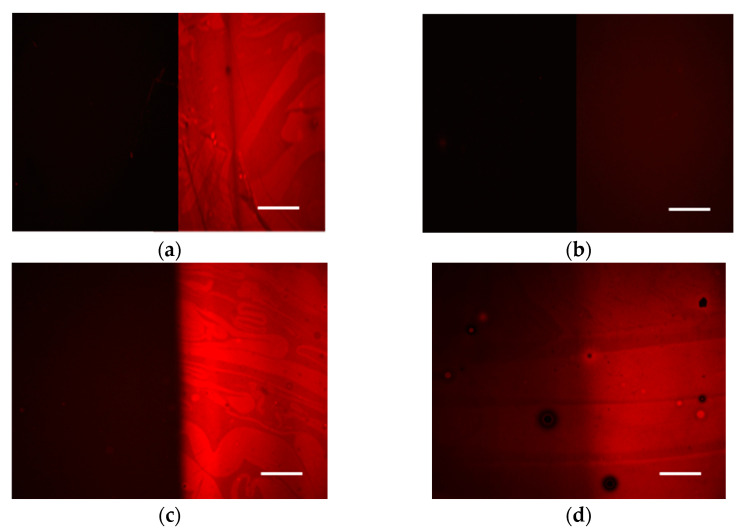
Fluorescence microscope mages received from silicon wafers coated with (**a**) PFMA homopolymer, (**b**) VGBE homopolymer, (**c**) PFMA:VBGE 1:1 copolymer, or (**d**) PFMA:VBGE 3:1 copolymer after incubation with biotinylated BSA and reaction with AlexaFluor546 labelled streptavidin. In all images, on the left is the unexposed area of the film and on the right is the exposed one. The scale bar corresponds to 50 μm.

**Figure 7:**
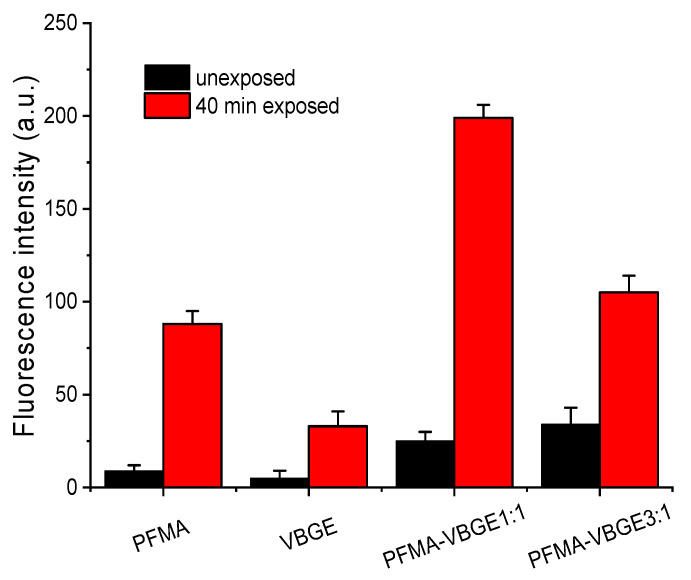
Net fluorescence intensity values obtained from the exposed (black columns) or the unexposed areas of the polymer films (red column) after adsorption of biotinylated-BSA and reaction with AlexaFluor546-labeled streptavidin.

The change in the water contact angle value of the exposed areas vs. the unexposed ones combined with the chemical changes in the surface could result in different behavior regarding their protein binding properties. Therefore, the exposed and unexposed areas of the polymers studied were incubated with a solution of biotinylated BSA and the relative adsorption of the protein to the two areas was estimated by measurement of the fluorescence intensity after reaction with a fluorescently labeled streptavidin. In Figure 6, characteristic fluorescence microscope images obtained from the unexposed and the exposed areas of the polymers, after biotinylated BSA immobilization and incubation with AF546-labeled streptavidin, are provided. The respective fluorescence intensity values are shown in Figure 7. The results show that for all polymers containing PFMA, their protein adsorption capacity increases when exposed to UV irradiation. This can be attributed to the cleavage of the pentafluorophenyl group and its replacement with a carboxylic acid, thus rendering the surface more hydrophilic, and with –COOH groups that facilitate the BSA adsorption mainly through hydrogen bonds and electrostatic interactions.

It is also worth mentioning that the two homopolymers as well as the PFMA:VBGE 1:1 copolymer have very distinct protein adsorption capacity between the unexposed and exposed areas, since all of them exhibit an approximately 10- to 15-fold higher fluorescence intensity in the exposed as compared to the unexposed area. Regarding the PFMA:VBGE 3:1 copolymer, a clear difference in the protein adsorption capacity between the unexposed and the exposed area is observed, as indicated by the measured 1:3 fluorescence intensity ratio between the unexposed and the exposed areas. In addition, the fluorescence intensity value corresponding to the unexposed area is the same as that obtained for the PFMA homopolymer despite the fact that the difference between the two areas was less pronounced compared to PFMA homopolymer and the 1:1 copolymer. A more throughout investigation with other proteins might help to conclude whether this is a general phenomenon for the particular copolymer or if it is also related with the nature of the adsorbed protein.

## 4. Conclusions

The ability to spatially control the properties of a surface is critical for many applications including the development of biomolecule arrays and biosensors. In the present work, two novel copolymers based on pentafluorophenyl methacrylate monomer (PFMA) were synthesized, exposed to UV light through a binary mask in presence of a photoacid generator, and baked to develop the pattern. It was found that: (a) the exposed areas of the polymer exhibited a reduction in their water contact angles values, except for the PFMA homopolymer, attributed to the cleavage of the pentafluorophenyl group from the ester group leading to a carboxylic acid group and the simultaneous cross-linking of the reacted epoxy groups creating hydroxyl groups, (b) both chemical changes in the exposed areas were verified by the change observed in the IR spectrum of the exposed and the unexposed areas as well as by ToF-SIMS analysis of the exposed and unexposed polymers surface, (c) the changes in the exposed areas resulted in enhancement of biomolecules adsorption in the exposed areas versus the unexposed ones, and (d) the latter was more pronounced for the PFMA homopolymer and the PFMA/VBGE 1:1 copolymer. Thus, the polymers and the simple patterning procedure developed based on them could be applied to create patterns of biomolecules onto surfaces altering their protein or cell binding properties in a spatially controlled mode. These surfaces can then be exploited either in bioanalytical applications such as the spatially selective immobilization of different biomolecules to biosensor chips for multiplexed determinations or to guided adhesion and growth of different types of cells on a 2D or 3D material for tissue engineering purposes.

## Figures and Tables

**Table 1:** Molecular characteristics of VBGE and PFMA homopolymers and copolymers ^a^.

Feed Ratio (%mol) ^a^ [PFMA]/[VBGE]	Composition (mol%) ^b^ [PFMA]/[VBGE]	M_n,SEC_^c^ (kg mol^−1^)	M_w_/M_n_^c^	Yield (%)
0/100	0/100	81.7	3.77	87
100/0	100/0	58.7 ^d^	3.14 ^d^	93
50/50	48/52	93.5	3.26	90
75/25	77/23	77.2	3.46	89

^a^ The (co)polymerization were initiated by AIBN at 70 °C in DMF. ^b^ Calculated by ^1^H NMR (CDCl_3_, 400 MHz). ^c^ Determined by SEC traces at 50 °C in DMF (1.0 mL min^−1^) using PSt standards. ^d^ Determined by SEC traces at 35 °C in THF (1.0 mL min^−1^) using PSt standards.

**Table 2:** Water contact angle (WCA) measurements of the homopolymers and the copolymers films in the unexposed and the exposed areas of the films. All contact angles given are the mean value of three measurements ± standard deviation.

Polymer	WCA Unexposed (°)	WCA Exposed 40 min (°)
PFMA	99 ± 4	98 ± 5
VBGE	66 ± 3	50 ± 4
PFMA/VBGE 1:1	84 ± 4	72 ± 3
PFMA/VBGE 3:1	94 ± 4	82 ± 4

## Data Availability

The data presented in this study are available on request from the corresponding author. The data are not publicly available due to privacy issues.

## Supplementary Materials

The following supporting information can be downloaded at: https://www.mdpi.com/article/10.3390/polym15030493/s1, Figure S1: (a) ^1^H NMR (400 MHz, CDCl_3_) spectrum and (b) ^13^C{^1^H} NMR (100 MHz, CDCl_3_) spectrum of the VBGE. Figure S2: (a) ^1^H NMR (400 MHz, CDCl_3_) spectrum. Asterisk (*) represents H_2_O. (b) SEC trace of poly(VBGE) homopolymer (M_n,SEC_ = 81.7 kg mol^−1^, M_w_/M_n_ = 3.77) received using as eluent DMF at a flow rate of 1.0 mL min−1 in a Agilent 1260 infinity system equipped with a 1200 HPLC pump, an Optilab T-rEX RI detector, and three 7.8 × 300 mm columns (Styragel^®^ HT 2, 3, and 4); Figure S3: (a) ^1^H NMR (400 MHz, CDCl_3_) spectrum of poly(PFMA) homopolymer. Asterisk (*) represents H_2_O. (b) SEC trace of the poly(PFMA) homopolymer (M_n,SEC_ = 58.7 kg mol^−1^, M_w_/M_n_ = 3.14) received using as eluent THF at a flow rate of 1.0 mL min^−1^ in a Viscotek GPCmax VE2001 system equipped with PSS columns (Styragel HR 3, 4 and 5); Figure S4: (a) ^1^H NMR (400 MHz, CDCl_3_) spectrum of poly(PFMA)-co-poly(VBGE) 3:1 copolymer. Asterisk (*) represents H_2_O. (b) SEC trace of the poly(PFMA)-co-poly(VBGE) 3:1 copolymer (Mn, SEC = 77.2 kg mol^−1^, M_w_/M_n_ = 3.46) received using as eluent DMF at a flow rate of 1.0 mL min^−1^ with a Agilent 1260 infinity system equipped with a 1200 HPLC pump, an Optilab T-rEX RI detector, and three 7.8 × 300 mm columns (Styragel^®^ HT 2, 3, and 4).; Figure S5: FTIR spectra of PFMA/VBGE 3:1 copolymer with PAG. Table S1: The water contact angle of PFMA:VBGE 1:1 and PFMA:VBGE 3:1 unexposed and exposed films without immersion of the films in solvents and when they are immersed in water, phosphate solution pH7.4 and hydrofluoroether (HFE 7500, from 3M Novec) for 1 h. Figure S6. Normalized ToF-SIMS signal intensities corresponding to the H_3_O^+^ ion of unexposed (black columns) or exposed areas (red columns) of PFMA homopolymer, VBGE homopolymer, PFMA/VBGE 1:1 copolymer and PFMA/VBGE 3:1 copolymer.
