# Supplementary material for: Post-Polymerization Modification of Fluoropolymers via UV Irradiation in the Presence of a Photoacid Generator
Source: Polymers (Basel). 2023 Jan 17;15(3):493. doi: 10.3390/polym15030493 (PMC9919986; doi:10.3390/polym15030493)
Supplement: Supplementary file 1 [file polymers-15-00493-s001.zip › polymers-2069715-supplementary.pdf]

polymers

MDPI

SUPPLEMENTARY MATERIAL

Post-polymerization modification of fluoropolymers via UV irradiation in the presence of a photoacid generator

Anastasia Nika [1], Christina Gkioka [1], Fotini Machairioti [1,2], Panayiotis Bilalis [3], Jiaxi Xu [3], Katarzyna Gajos [4], Kamil Awsiuk [4], Panagiota Petrou [2,*] and Margarita Chatzichristidi [1,*]

1 Industrial Chemistry Laboratory, Department of Chemistry, National and Kapodistrian University of Athens, Panepistimiopolis Zografou, 15771, Greece
2 Immunoassay/Immunosensors Lab, INRaSTES, NCSR "Demokritos", Aghia Paraskevi, 15310, Greece
3 Polymer Synthesis Laboratory, KAUST Catalysis Center, Physical Sciences and Engineering Division, King Abdullah University of Science and Technology (KAUST), Thuwal 23955, Saudi Arabia
4 M. Smoluchowski Institute of Physics, Jagiellonian University, Łojasiewicza 11, 30-348 Kraków, Poland

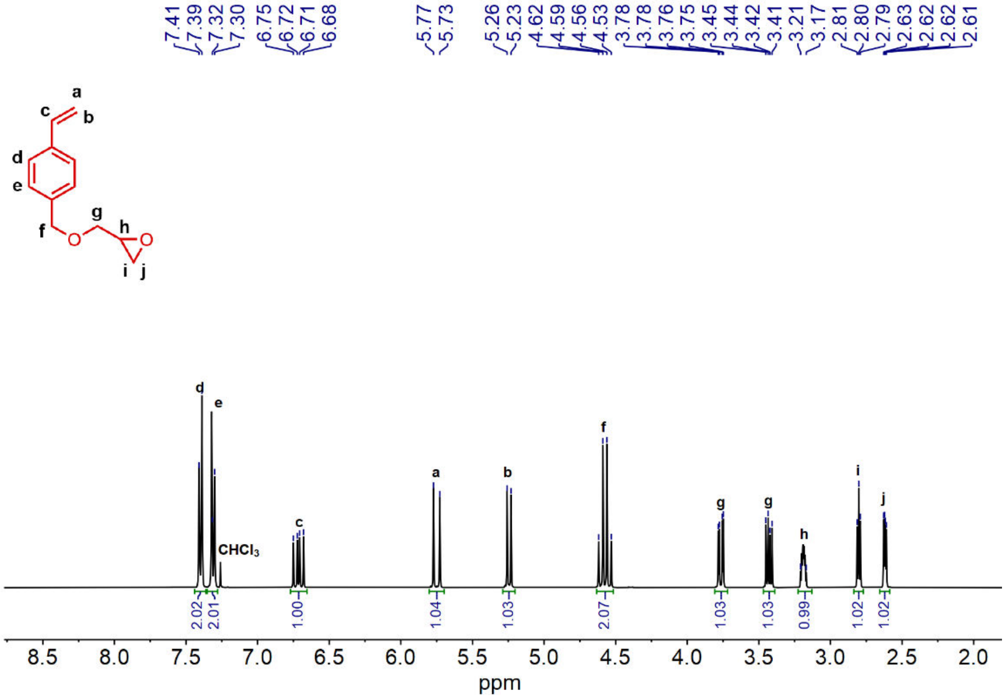

(a)

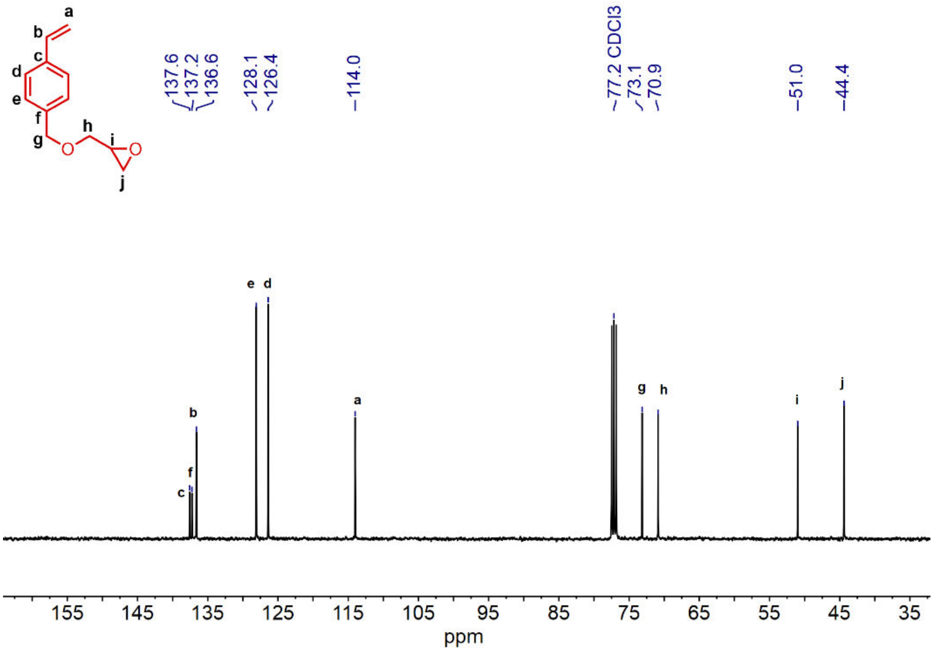

(**b**)

Figure S1. **(a)** ^{1}H NMR (400 MHz, $CDCl_3$) spectrum and **(b)** $^{13}C\{^{1}H\}$ NMR (100 MHz, $CDCl_3$) spectrum of the VBGE.

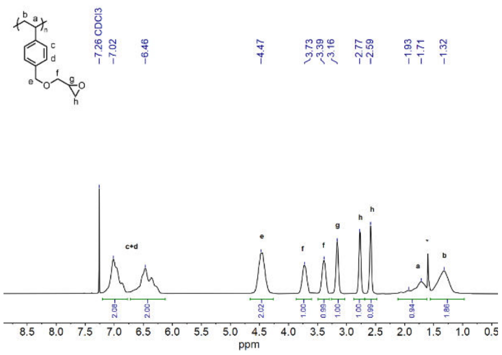

(**a**)

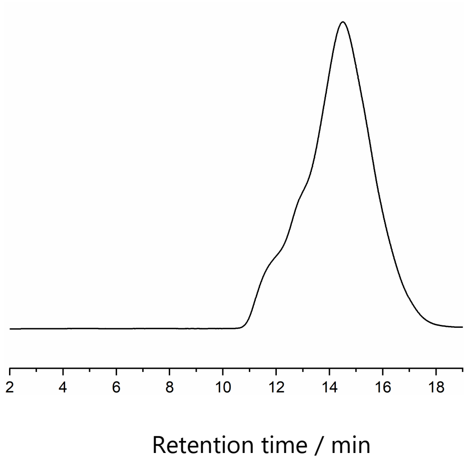

(**b**)

Figure S2. (a) ^{1}H NMR (400 MHz, $CDCl_3$) spectrum of poly(VBGE) homopolymer. Asterisk (*) represents H_2O. **(b)** SEC trace of poly(VBGE) homopolymer ($M_{n,SEC}$ = 81.7 kg mol^{-1}, M_w/M_n = 3.77) received using as eluent DMF at a flow rate of 1.0 mL min^{-1} in an Agilent 1260 infinity system equipped with a 1200 HPLC pump, an Optilab T-rEX RI detector, and three 7.8 × 300 mm columns (Styragel® HT 2, 3, and 4).

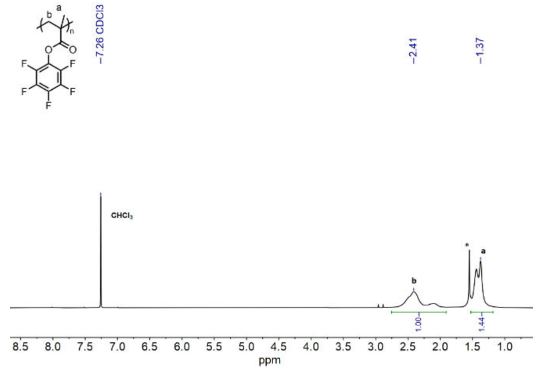

(a)

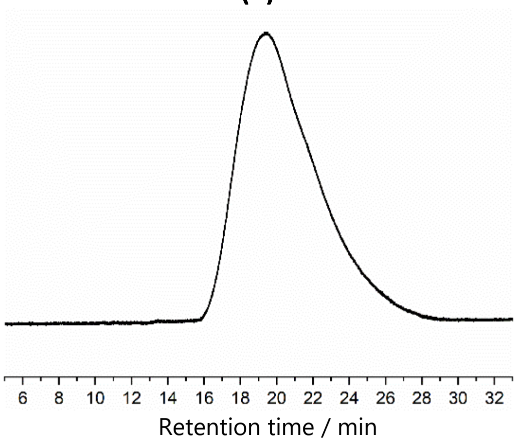

(b)

Figure S3. (a) ^{1}H NMR (400 MHz, $CDCl_3$) spectrum of poly(PFMA) homopolymer. Asterisk (*) represents H_2O. **(b)** SEC trace of the poly(PFMA) homopolymer ($M_{n,SEC}$ = 58.7 kg mol^{-1}, M_w/M_n = 3.14) received using as eluent THF at a flow rate of 1.0 mL min^{-1} in a Viscotek GPCmax VE2001 system equipped with PSS columns (Styragel HR 3, 4 and 5).

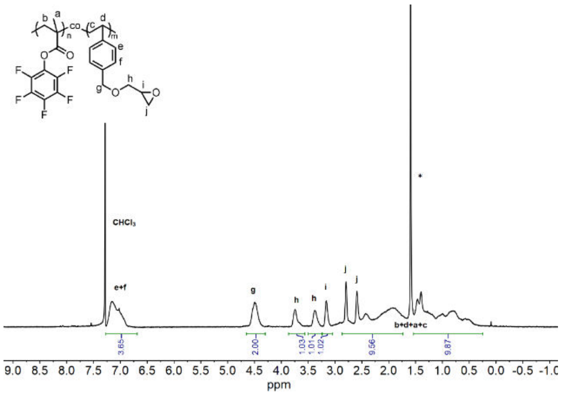

(a)

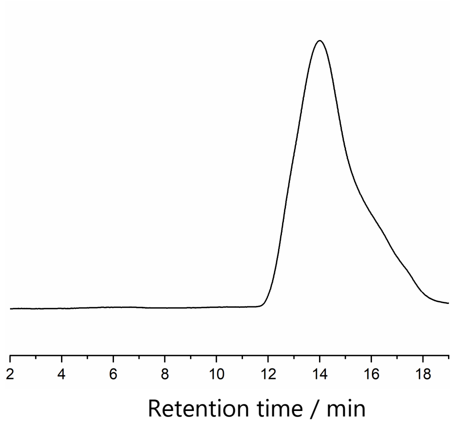

(b)

Figure S4. (a) ^{1}H NMR (400 MHz, $CDCl_3$) spectrum of poly(PFMA-co-VBGE) 3:1 copolymer. Asterisk (*) represents H_2O. **(b)** SEC trace of poly(PFMA-co-VBGE) 3:1 copolymer ($M_{n,SEC}$ = 77.2 kg mol^{-1}, M_w/M_n = 3.46) received using as eluent DMF at a flow rate of 1.0 mL min^{-1} with an Agilent 1260 infinity system equipped with a 1200 HPLC pump, an Optilab T-rEX RI detector, and three 7.8 × 300 mm columns (Styragel® HT 2, 3, and 4).

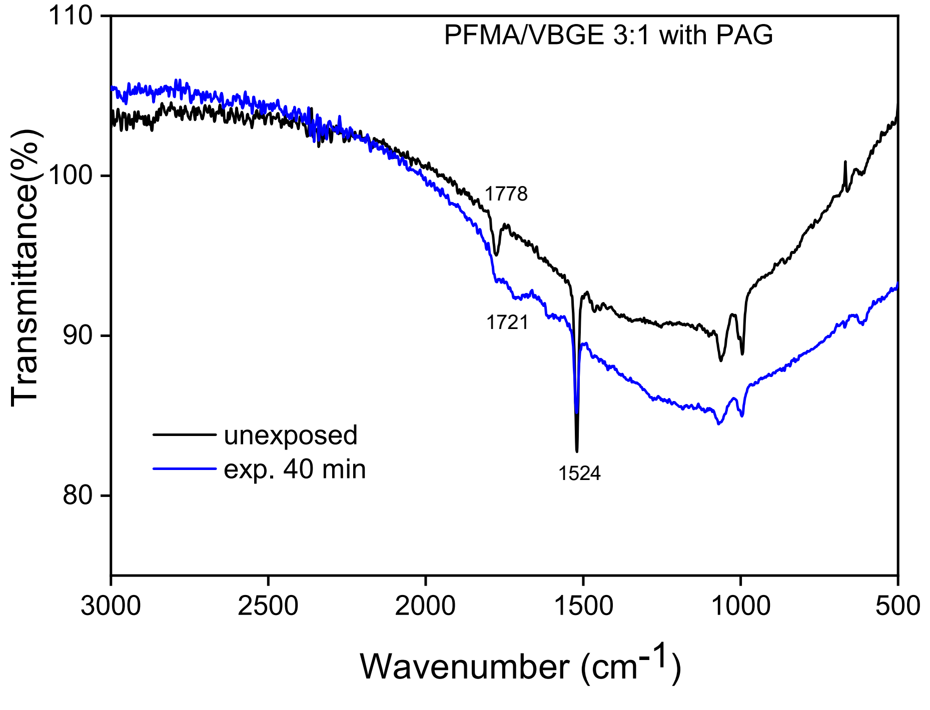

Figure S5. FTIR spectra of PFMA/VBGE 3:1 copolymer resist prior to (black line) and after 40-min UV exposure (blue line).

Table S1. The water contact angle of PFMA:VBGE 1:1 and PFMA:VBGE 3:1 unexposed and exposed films without immersion of the films in solvents and when they are immersed in water, phosphate solution pH7.4 and hydrofluoroether (HFE 7500, from 3M Novec) for 1 hour.

**Immersion solvent**	**Contact Angle (⁰)**	
**PFMA:VBGE 1:1**	**Unexposed**	**Exposed**
None	84	71

Water	71	65
PBS 7.4	60	45
HFE	86	103
**PFMA:VBGE 3:1**		
None	94	82
Water	85	73
PBS 7.4	76	52
HFE	96	101

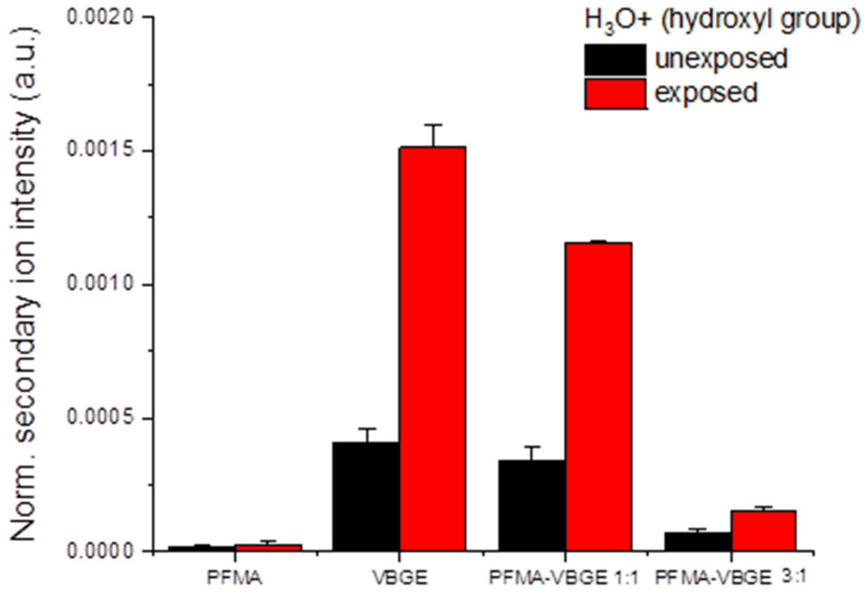

Figure S6. Normalized ToF-SIMS signal intensities corresponding to the H_3O+ ion of unexposed (black columns) or exposed areas (red columns) of PFMA homopolymer, VBGE homopolymer, PFMA/VBGE 1:1 copolymer and PFMA/VBGE 3:1 copolymer.
